# Women Consumers’ Views on Legislation to Restrict Prominent Placement and Multibuy Promotions of High Fat, Sugar, and Salt Products in England: A Qualitative Perspective

**DOI:** 10.34172/ijhpm.2023.7597

**Published:** 2023-09-10

**Authors:** Preeti Dhuria, Sarah Muir, Wendy Lawrence, Emma Roe, Sarah Crozier, Cyrus Cooper, Janis Baird, Christina Vogel

**Affiliations:** ^1^Medical Research Council Lifecourse Epidemiology Centre, University of Southampton, Southampton General Hospital, Southampton, UK; ^2^National Institute for Health Research Southampton Biomedical Research Centre, University of Southampton and University Hospital Southampton NHS Foundation Trust, Southampton, UK; ^3^School of Geography and Environmental Science, University of Southampton, Southampton, UK; ^4^NIHR Applied Research Collaboration Wessex, Southampton Science Park, Innovation Centre, Southampton, UK; ^5^Centre for Food Policy, City, University of London, London, UK

**Keywords:** Supermarket Environment, Obesity, UK Food Policy, HFSS Regulations, Food Shopping Behaviours

## Abstract

**Background:** As part of the childhood obesity strategy, the UK Government has introduced regulations to restrict the ways high fat salt and sugar (HFSS) products can be promoted in retail settings from October 2022. This study explored (*i*) consumers’ views on the likely impact of the UK legislation restricting the placement and promotion of HFSS products on their shopping behaviours and (*ii*) consumers’ beliefs about who is responsible for healthy eating.

**Methods:** Using a cross-sectional study design, qualitative semi-structured telephone interviews were conducted with a purposive sample of women who shopped at a discount supermarket. Thematic analysis was employed to identify key themes.

**Results:** Participants’ (n = 34) had a median age of 35 years and over half were in paid employment. Five themes were identified: (1) The legislation is acceptable, but people can still (and should be able to) buy HFSS items; (2) The legislation is likely to have more impact on shoppers who do not plan their shopping; (3) Affordability of healthy food is just as, or more, important than the legislation; (4) It’s up to the individual to eat healthily; and (5) Government and retailers can better support consumers to make healthy choices.

**Conclusion:** Most participants were optimistic about the incoming regulations and believed that it would support consumers to make healthier food choices. Many raised concerns, however, that the high price of healthy foods and continued availability of unhealthy foods within the stores could undermine the legislation’s benefits. Coupling the legislation with interventions to promote and reduce the costs of healthier products would go some way to ensure its success. Raising awareness about marketing strategies that play into consumer concerns for cost and autonomy could further increase acceptance of the policy.

## Background

**Key Messages**

**Implications for policy makers**

- The unaffordability of healthier foods could undermine the high fat salt and sugar (HFSS) placement and promotion legislation’s benefits and be detrimental for low-income families.
- Consumers believe in autonomy of their food choices, and many may continue to purchase the less healthy foods that are available.
- Coupling the legislation with interventions to promote and reduce the costs of healthier products would go some way to ensure its success.
- Families with the lowest food budgets tend to plan their shopping in detail, therefore evaluations of the legislation should assess impact across a range of sociodemographic groups and on health inequalities.
- Implementing initiatives to raise consumer awareness about how marketing strategies restrict consumer choice and lead to increased spend on HFSS could help to increase consumer support for the policy.

**Implications for the public**

This study explored consumer’s perceptions towards the incoming food placement and promotion legislation and how it is likely to impact their food shopping behaviours. Most considered the legislation would reduce impulse buying of less healthy foods but acknowledged that the higher price of healthier foods could limit the benefits. Consumers who plan their shopping find it easier to buy fewer unhealthy foods and many may benefit from learning more about how marketing practices encourage unhealthy food choices.

High intake of foods containing elevated levels of saturated fat, free sugars and salt is associated with increased prevalence of non-communicable diseases such as obesity, cardiovascular disease and type-2 diabetes which is costly to society and individuals.^1^ The greatest burden of disease falls upon families experiencing socioeconomic disadvantage and the COVID-19 pandemic has exacerbated existing inequalities, doubling the deprivation gap in childhood obesity.^2^ Such inequalities result from intergenerational patterning of environmental, social, economic and behavioural determinants of dietary quality.^3,4^ Government regulation is one of the most efficient, cost-effective, and far-reaching ways to change the food infrastructure which may positively contribute towards the health of individual consumers.^5^

 From October 1, 2022, the UK Government implemented legislation restricting the prominent placement (ie, store entrances, aisle ends, and checkouts) of high fat salt and sugar (HFSS) products in retail outlets and their online equivalents.^6,7^ Plans to restrict multi-buy promotions on HFSS products have been delayed until October 2025 due to economic pressures on families and businesses as the cost of living increases.^8^ To our knowledge, this comprehensive legislation is one of the first in the world to restrict where retailers can position less healthy food products and forms part of the UK government’s strategy to tackle childhood obesity.^9^ Marketing strategies such as prominent product placement and price promotions are commonly used to promote less healthy food which in turn influences consumers’ choices and purchasing behaviours.^10,11^ There is increasing scientific evidence to demonstrate that healthier placement initiatives can shift food sales to be more nutritious, proving support for this government action.^12,13^

 The healthfulness of retail settings can be determined using tools that assess the in-store environment by measuring the availability, price, promotions, placement, variety, quality, and/or nutrition information on a selection of healthy (eg, fruit, vegetables, high fibre cereals and breads, etc) and less healthy products (eg, crisps, confectionery, sugary drinks, white bread, processed meats etc).^14^ These less healthy stores thereby compound existing inequalities in food choice because more socioeconomically vulnerable consumers have fewer psychosocial, financial and educational resources to protect them against unhealthy contextual cues.^15-17^ Our previous qualitative work with women who shopped in less healthy discount stores,^18^ revealed that their intended shopping choices were frequently undermined by the prominent placement and promotion of unhealthy foods.^14,19^ The incoming placement and promotions legislation therefore has potential to reduce dietary inequalities.

 This is the first national legislation to restrict the promotion and placement of a number of different product categories and it is unknown how consumers will react, and what intended and unintended consequences it may have on their shopping practices. Understanding consumers’ perspectives will provide raw insights into adaptations they may make to their shopping behaviours or reveal whether further strategies to ensure public support are needed; these insights could inform effective implementation and uncover the potential public health impact the policy could have. Thus, rather than being driven by theory, this study adopted an inductive approach to understand consumers’ beliefs about the legislation’s potential impact. Furthermore, understanding the views of consumers themselves about who is responsible for healthy eating (ie, individuals, government, businesses) could also provide insight into factors influencing the acceptability and effectiveness of this incoming legislation. Using a sample of women who shopped at less healthy supermarkets, this qualitative study aimed to answer the following research questions: (*a*) what impact do consumers think the food placement and promotion legislation is likely to have on food shopping behaviours? (*b*) who do consumers believe is responsible for healthy eating?

## Methods

###  Setting and Ethics

 This study forms part of the process evaluation activities of the WRAPPED (Women’s Responses to Adjusted Product Placement and its Effects on Diet) study which is a natural experiment conducted in collaboration with a UK discount supermarket chain. WRAPPED utilises a prospective matched controlled cluster design to examine the impact of improved product placement of healthy foods in stores on the purchasing and dietary patterns of women and their young children.^20^ The intervention involved increasing the availability of fresh fruit and vegetables, and placing them towards the front of stores. A control store was matched to each intervention store with improved layout on the basis of: (*i*) sales profile, (*ii*) customer profile, and (*iii*) neighbourhood deprivation (index of multiple deprivation, IMD)^21^ and was distanced at least 20 miles from an intervention store to reduce contamination of data. Discount supermarkets were chosen as the setting for this study because they typically have less healthy in-store environments than mid-range and premium supermarkets, having poorer availability, pricing and placement of healthy foods, and favouring promotion of unhealthy foods.^14^ This study abides by the Declaration of Helsinki, Research Governance Framework for Health and Social Care and Data Protection regulations. Reporting of this study follows Consolidated criteria for Reporting Qualitative research (COREQ) recommendations.^22^

###  Participants

 A purposive sample of 40 participants from both intervention and control stores of the WRAPPED study were invited to take part in this qualitative study. Aligned with the study protocol, participants of the WRAPPED study were aged between 18-45 years, held a loyalty card at the collaborating discount supermarket, and had shopped in a study store during the 12 week period prior to recruitment. The sampling frame aimed to include approximately equal representation from the WRAPPED intervention and control stores, north and south English regions, low and higher education levels, and households with and without young children. Information about demographic characteristics was collected, including participants age, ethnicity, marital status, neighbourhood deprivation (IMD), living with children, employment status and money spent on groceries each week. Educational attainment was used as a proxy for socioeconomic status asit is one of the strongest markers of dietary quality,^23^ shapes other socio-demographic markers including employment status, job type and income^24^ and higher educational attainment can help protect against exposure to unhealthy supermarket environments.^17^ Invitation letters were sent by post and an incentive (£10 voucher) was offered. Interested participants contacted the research team to schedule a telephone interview. Two women (not study participants) aged between 18 and 45 years, who shopped at the collaborating discount supermarket and held a store loyalty card were recruited to our WRAPPED Patient and Public Involvement (PPI) panel via targeted Facebook adverts. They provided valuable insight to the design, methods and interpretation of findings throughout the WRAPPED study.^20^

###  Interviews

 The semi-structured interview guide (see Supplementary file 1) was devised to ask questions about (*i*) shopping habits during COVID-19, (*ii*) participants’ experiences of how retail promotions and placement strategies influence their food choices and perceptions of the food (placement and promotion) legislation, and (*iii*) beliefs about government, retailer, and individual responsibility for healthy eating. Participants responses to the questions regarding experiences of retail promotions and placement strategies were asked to orientate participants to the topic area of interest in this study. These data were not specifically analysed in this study because we have previously published data describing such lived experience.^19^ Therefore, the analyses in this paper focused on questions related to perceptions to the incoming government regulation and responsibility for healthy eating. The full interview schedule was pilot tested with our WRAPPED PPI representatives after which minor edits were made to the wording of questions. Using a semi-structured interview guide allowed topics of interest to be explored systematically and comprehensively, while still enabling participants to direct the discussion and raise specific issues relevant to them.^25^ Interviews were conducted by phone and audio-recorded following participant’s consent. All interviews were conducted by PD, a registered public health nutritionist with over 10 years’ experience in health research. Some participants may have spoken to PD during WRAPPED outcome data collection but for most participants this was their first interaction with her.

###  Thematic Analysis 

 After removing all personal details, audio-recordings were transcribed verbatim. The data were analysed using inductive thematic analysis and guided by the research questions exploring likely impact of legislation on food shopping behaviours and responsibility for healthy eating.^26^ SM, a psychologist with over 15 years’ experience conducting qualitative analysis, identified the initial themes. SM had little prior knowledge of consumer shopping styles and food policy. Each transcript was read for familiarisation and a summary of main points made by each participant was used to create initial codes. Using Microsoft Excel, key points were collated into broad, overarching themes relating to: shopping style, acceptability of legislation, impact of legislation, and suggestions for change. The Excel spreadsheet became refined over time to include quotes that related to emerging sub themes. The spreadsheet had a row of data for each participant which also included columns indicating education attainment, control or intervention group and weekly shopping budget (categorised as very high, high, average, low, very low based on comparisons to the national average). This approach allowed for comparison of themes to be made against these participant characteristics. Overarching themes were then reviewed and analysed in detail to understand nuances in meaning and were discussed at length by PD, SM, and CV before the final themes were agreed. SM and PD each reanalysed 10% of the transcripts and discussed any disagreements – these required only small changes to theme descriptions for clarity. The final themes and interpretation of these themes, including relationships between them, were discussed with our PPI representatives.

## Results

 A total of 34 women expressed interest and consented to participate. They were interviewed in May 2020 and there were no dropouts; interviews lasted between 17 and 48 minutes (mean = 32 minutes). Slightly more participants (62%) were from the WRAPPED control stores^20^ and were in paid employment (57%) and half had no educational qualifications beyond those attained at age 16 years (Table). Medians and interquartile ranges are provided for age and pounds spent per week on food. Percentages are provided for all other variables. Differences in medians for non-normally distributed continuous variables (age, educational qualifications, IMD decile and money spent on food per week) were assessed using Mann-Whitney rank sum tests. Differences in percentages for categorical variables (ethnicity, marital status, employment status, and living with children) were assessed using chi-squared tests. Median (interquartile range, IQR) participant age was similar in the control group (35.7 [31.7, 39.1] years) and intervention group (35.9 [32.4, 39.7] years), *P* =.45. In the control group 76% of women were of white ethnicity, compared to 85% of women in the intervention group (*P* =.56).

**Table:** Participant Demographic Characteristics

**Characteristic**	**Total** **(n = 34)**	**Control** **(n = 21)**	**Intervention ** **(n = 13)**	***P*** ** Value**
Age (y), median (IQR)	35.7 (31.7, 39.4)	35.7 (31.7, 39.1)	35.9 (32.4, 39.7)	.45
White ethnicity, % (n)	79% (27)	76% (16)	85% (11)	.56
Married, % (n)	61% (19)	67% (12)	54% (7)	.44
Low education (no qualifications beyond age 16), % (n)	50% (17)	62% (13)	31% (4)	.49
Most deprived half of area deprivation (IMD), % (n)	29% (10)	29% (6)	31% (4)	.58
Paid employment, % (n)	57% (19)	55% (11)	62% (8)	.71
Pounds (£) spent on food per week, median (IQR)	70 (45, 100)	70 (50, 100)	80 (40, 90)	.76
Percentage (number) with children in the household	82% (27)	83% (10)	82% (27)	.87

Abbreviations: IQR, interquartile range; IMD, index of multiple deprivation. Mann-Whitney rank sum tests and chi-square tests.

 Five core themes were identified in relation to the research questions: three related to the first question and two related to the second. No differences in views by demographic characteristics were identified in the thematic analysis. Each of these themes are discussed in detail below, accompanied by illustrative participant quotes. Details of whether participants were from the control (Cont) or intervention (Int) stores and low (LE) or higher (HE) education level are indicated.

###  Research Question 1: What Impact Do Consumers Think the Food Placement and Promotion Legislation Is Likely to Have on Food Shopping Behaviours ?

####  Theme 1: The legislation is acceptable, but people can still (and should be able to) buy HFSS items if they want them. 

 Most participants responded positively to the legislation, believing it would help improve population health and address obesity by encouraging people to make healthier food choices. Participants believed that fewer promotions on HFSS items would make them less appealing, reduce pressure from children and other family members, and discourage customers from buying more HFSS items than they had planned to.

 “*I think the intent is good so that it discourages people from buying, especially the location bit like sometimes they buy it because they can see it […] if there’s no promotion at all then I guess we will be forced to reduce our spending on unhealthy food which may be a good thing*” (P6280, Int, HE).

 “*I think it is good. I think they should restrict it […] if they are trying to get everyone to be healthier then they shouldn’t be putting things on the shelves, or you know at the end of tills or at the front to encourage people to eat that bad thing*” (P6297, Cont, LE).

 Some participants felt less positive about the legislation, believing the government should not be interfering, or expressed concerns about the effectiveness of the legislation given that consumers were still able to buy HFSS products in-aisle or from other retailers.

 “*I’m kind of split because in a way government should butt out, what people pick is their own choice. But in another way with obesity epidemics and obesity in children and stuff like that I would probably say yeah great, so it’s a split one that one*” (P6298, Cont, HE).

 “*I think its nanny state, I think it’s ridiculous. I just think if people want to buy the high fat, the high sugar foods people will buy them anyway so leave them to it*” (P6275, Cont, LE).

####  Theme 2: The legislation is likely to have more impact on shoppers who do not plan their shopping.

 Participants discussed their shopping styles and practices, which highlighted a likely differential impact of the legislation according to various shopping behaviours. Some participants described approaching their food shopping in a very conscious, planned manner which involved checking ingredients at home, meal planning and following a shopping list. Participants who planned before food shopping described making fewer impulse purchases or overbuying. Planning food shopping was often associated with participants sticking to a diet plan (eg, Slimming World), health being important to them or to accommodate a tight budget. However, some planned shoppers on universal credit reported buying unhealthy meal deals and predominantly freezer food which is suggestive of having a poor dietary pattern.

 “*I come up with kind of ideas for meals for the week and make a list of all the ingredients I would need […] I actually just look for the product so it’s a specific thing that I’m looking for so I will just look for that item no matter, you know regardless of where it’s placed. I think that’s the difference I’ve found from you know browse shopping to actual like target shopping if that makes sense*” (P6266, Int, HE).

 “*I have to buy and survive for the month so normally I know what I’m going in for, I get it and I go*” (P6282, Int, LE).

 Women who planned their food shopping reported that the legislation will have limited impact on their food choices. These consumers will still buy some HFSS products as treats, in moderation but the restrictions on the marketing of these foods is likely to have little impact on them.

 “*I don’t think it [legislation] will affect my shopping habits like I do buy them, but it is not something we sit and eat all the time, it is given to the kids as a treat, so I mean unless you cannot buy them at all I don’t think it is really going to affect me*” (P6302, Cont, LE).

 Families who plan their shopping to a very tight budget do use price promotions, but these are consciously planned by comparing deals across supermarkets and visiting multiple retailers to buy deals or occasionally buy more-expensive branded products. Those with larger families and who rely on promotional strategies, felt they were likely to be impacted negatively by the legislation.

 “*I always look for good brands, a good brand but on rollback. I feel then that I’ve got a quality product. My aim is to get as much as I can for spending less. To make the money go further as a family*” (P6054, Int, HE).

 “*If it’s [HFSS products are] too expensive obviously with having so many of them [5 children] then I don’t buy it. So yeah, it’s going to [affect] parents that rely on those mulitbuys and promotions and stuff like that*” (P6298, Cont, HE).

 In contrast, participants who did not plan their shopping reported being more likely to be tempted by items in prominent locations and choose foods simply because they were on promotion. Women who did not plan their food shopping are likely to be positively impacted by legislation.

 “*Yeah, I’m a sucker for that, I do buy promotion stuff like two for one and stuff. When you go straight through the door and you see it then you think oh that’s a good deal and then just pick it up and put it in the trolley and then when you get to the till you see the stuff there, then you just pick that up as well” *(P6314, Cont, LE).

####  Theme 3: Affordability of healthy food is just as, or more, important than the legislation.

 Some participants expressed concern about the affordability of products for those who are accustomed to regularly buying items on promotion or that the money previously saved by consumers would now add to retailers’ profits.

 “*There’s a lot of people that look out for promotions, because a lot of people can’t afford them in the first place. So that’s their way of making something last them longer or buying probably twice at one time because they might not be able afford it the following week*” (P6030, Int, HE).

 “*So instead of the savings being you know sent back down to the customer it’s going to just be money in the pocket of the Corporations isn’t it*” (P6266, Int, HE).

 Participants also felt the legislation would have greater impact on population health if it was implemented in conjunction with increased promotion and reduced cost of healthier options, particularly fruit and vegetables. Many women commented that healthy foods were considerably more expensive than unhealthier options which restricted their ability to follow a healthy diet and made it easier to choose HFSS products.

 “*No point in them [Government] discouraging the non-healthy food when the healthy food prices are going really really high*” (P6301, Cont, LE).

 “*I do strongly believe the government should actually keep the price of higher sugar and fat foods higher and the healthier food should be lower in price. Because at the moment it’s the opposite and that’s why a lot of people are finding it easier to just eat the bad food and that’s how we have a lot of obesity cases because it is cheaper to be bigger than slimmer*” (P6303, Cont, HE).

 Participants also commented that cheaper HFSS products last longer and can be used for snacks throughout the week compared to more expensive healthier products which may comprise only a single, family snack. Women stated that the price of healthier food needed to be reduced and suggested that supermarkets offer more price promotions and permanent price reductions on fruits, vegetables, and healthier snacks to encourage long-lasting dietary change and allow parents to regularly buy these options for their children.

 “*I do feel with the crisps and snacks you do get more for less so obviously it does stretch out […] A punnet of strawberries can be £3, and they last one day. Whereas a packet of crisps can last all week*” (P6302, Cont, LE).

 “*I find it frustrating that the unhealthy food are the cheaper ones and that I could buy rubbish for nothing and that I have to invest in making healthier choices. For mothers who simply don’t have the money it’s a choice between that, or putting gas and electricity on, and that’s a horrible position to be in*” (P6101, Cont, HE).

###  Research Question 2: Who Do Consumers Believe Is Responsible for Healthy Eating? 

####  Theme 4: It’s up to the individual to eat healthily: the importance of consumer autonomy.

 Participants, regardless of education level or budget, consistently expressed the view that consumers are ultimately responsible for what they choose to buy and eat. Many stated that it is their own decision whether or not they make healthier or unhealthier choices.

 “*You chose what you want to buy. No one is forcing you to have it regardless of the marketing. As an adult you are fully aware of what you are choosing to put in the trolley and pay for*” (P6302, Cont, LE).

 “*If you’re paid monthly and you’re coming to the end of that month and you’ve got nothing, that’s your problem, you should’ve budgeted better*” (P6275, Cont, LE).

 “*I think the onus has to be on the person really, the individual themselves to make that decision [to buy healthier foods], the shops at the end of the day are a business they want to make a profit*” (P6039, Cont, HE).

 The strong belief that participants had about the importance of consumer autonomy meant they felt there was a limit to how extreme government intervention could be.

 “*I don’t think the government can police, or be legislative about the choices that people make, but they can encourage a culture where we value health and wellbeing, where we are encouraged to take our own individual responsibility which includes keeping as healthy as possible for the NHS*” (P6101, Cont, HE).

 “*The government and all shops can do all the help and offer it to everybody but if people don’t want to do it there’s nothing, they can do but I think they should just try and put the healthy foods out there*” (P6282, Int, LE).

####  Theme 5: Government and retailers can better support consumers to make healthy choices.

 Although participants held strong views about personal responsibility for food decisions, they also made suggestions for how supermarkets and government could support them to make healthier choices. The difference in price between healthy and less healthy foods was raised again. While some participants believed that individuals are responsible for ensuring they budget appropriately to be able to afford healthy food as discussed in theme 4, many other participants felt that supermarkets were responsible for ensuring healthy food was affordable or that governments could offer incentives/vouchers to guarantee healthy foods are accessible for all consumers.

 “*By making it (healthier food) a lot more cheaper and a lot more special offers, and if they’re families then they (government) could always offer them like vouchers or something to help promote them to eat healthy…” *(P6303, Cont, HE).

 Participants were pleased with government’s actions on taxing sugary drinks and felt that reduced promotion of less healthy foods and increased promotion of healthy products in advertising, media and at bus stops was needed.

 “*I think it’s good that they brought in things like the sugar tax and that sort of thing. I think there should be tax on unhealthy things, I think there should be tax on alcohol, cigarettes, sugar, takeaways, and that sort of things because that would encourage people to maybe not buy them as much” *(P6308, Cont, LE).

 “*Like they did with the sugar they put it up in price and I think they should put the unhealthier stuff up in price slightly. Then that would encourage people to then eat healthier. And have not so many adverts on the television, regarding unhealthy stuff” *(P6306, Int, HE).

 Other suggestions for how supermarkets could better support healthier choices included moving fruit, vegetables, healthier snacks, and healthy meal ingredients to prominent locations in place of HFSS products, having price promotions on healthy meal options, and co-locating healthy options with standard/unhealthy products.

 “*I think by putting the healthier things nearer the front would help, because some people might only be nipping in for a couple of things” *(P6054, Int, HE).

 “*I think they should try and put better promotions on the healthy foods” *(P6285, Cont, HE).

 “*I think they could do more deals at the end of the aisles instead of it being chocolate and cakes” *(P6101, Int, HE).

## Discussion

###  Principal Findings

 Participants in the current study were generally positive about the incoming legislation and the impact it could have on reducing impulse buying of HFSS and improving population health. However, they also provided insight into a number of potential unexpected consequences of the legislation which have important implications for its effectiveness, evaluation, and acceptability. In particular, participants described: (*i*) that the unaffordability of healthier foods could undermine the legislation’s benefits and be detrimental for low-income families, (*ii*) differential impact of regulation according to shopping styles/practices (ie, planned vs unplanned food choices), and (*iii*) because consumers have autonomy in their food choices and HFSS items will still be available to buy, many may simply change their journey through the shopping environment and not alter their purchasing patterns. Participants suggested ways in which both retailers and the government could support consumers in making healthier purchases.

###  Comparison With Previous Literature

 Women participating in this study generally expressed their support for the incoming legislation to limit the prominent placement and promotion of HFSS products in retail outlets, although some believed it threatened individuals’ freedom to make food choices. These findings are similar to research conducted with parents of young children following the introduction of UK Soft Drinks Industry Levy in 2018, a volume tax based on the sugar concentration of non-alcoholic drinks.^27^ A large international survey conducted across five counties revealed that public support for supermarket placement and availability interventions in the United Kingdom was moderate compared to the four other countries (Australia, the United States, Canada, and Mexico).^28,29^ Survey responses from the United Kingdom showed the greatest public support for subsidies to reduce the price of fresh fruit and vegetables and for initiatives increasing the availability/shelf space of healthier options.^28,29^ Furthermore, socioeconomically disadvantaged families were less likely to favour legislative nutrition policies than more affluent participants. This finding may be attributed to the perception that these interventions limit individual choice and favour the narrative of individual responsibility for making healthier food decisions. Our study findings suggest that socioeconomic status may not predict support for legislative nutrition policies alone but the difference in results may relate to the qualitative approach used in the current study.

 This study also suggests that adopting a planned shopping style could be protective against in-store marketing strategies that promote HFSS products. Previous research indicates that meal planning is associated with a healthier diet and lower levels of obesity.^30,31^ Dubowitz et al demonstrated that poorer families who consistently used a shopping list have better quality diets and were less likely to experience unhealthy bodyweight than shoppers who did not plan their purchases.^31^ These findings suggest that while interventions to enhance meal-planning behaviours may be a tool for consumers to navigate unhealthy shopping environments, this approach requires consumers to be highly motivated.^32^ For the participants of the current study, using a shopping list was motivated by being health conscious, wanting to reduce food waste and/or needing to adhere to a very restricted grocery budget.

###  Policy and Research Implications 

 Evaluation of the incoming legislation should be designed to assess possible unanticipated consequences due to differential shopping styles and affordability of healthier foods, some of which have been highlighted in this study. In particular, families most socioeconomically disadvantaged may be minimally affected by the legislation because of their strict adherence to shopping lists which are planned to enable their food budget to stretch as far as possible. However, the legislation may be highly effective among families who do not actively plan their shopping purchases. These differences in shopping practices could have implications for dietary inequalities, with those with the poorest quality diets receiving little benefit of the legislation without additional financial support. Most participants who reported low or very low grocery spend described planning their food shopping trips to achieve best value for their money. Although planning can be protective to navigate retail settings, it is not necessarily sufficient to support healthier consumption. The legislation is likely to be maximally effective if implemented alongside interventions that make fruit and vegetables more affordable and appealing, and ensure consumers have the time and resources required to plan, prepare, and cook healthier meals.^33,34^

 The higher price of healthy foods, compared to HFSS foods, was raised by many participants as a factor that could undermine effectiveness of the incoming legislation. Healthy food has been shown to cost more than unhealthy food per calorie and less money is spent on marketing healthy foods by businesses.^35^ These factors make purchasing healthy foods restrictively expensive not an attractive option and not good value for lower income families. Recent research revealed that high fixed costs in the supply chain play a much greater role in the price of fruit and vegetables than the cost of other foods, meaning consumers buy approximately 15% fewer fresh fruit and vegetables than they would have if these retail market imperfections were removed.^36^ The economists conducting the study recommend the UK government introduce a 25% subsidy on fruit and vegetables to correct the market and improve population diet.^36^ Evidence shows that retailers can employ temporary price promotions and prominent placement of an expanded range of fruit and vegetables to improve population purchasing behaviours,^37,38^ but further evidence of the effect of these strategies at a household and individual level and on inequalities is needed.^20^

 Findings from this study and previous literature show that women who shopped at discount supermarkets attribute ultimate responsibility for healthy eating to themselves^19^ and do not recognise the powerful influence of food marketing practices and how they undermine personal choice. This sense of individual responsibility likely stems from the need to exercise autonomy over one’s life, including food choices. Autonomy is the capacity to make informed choices in relation to oneself and responsibility is the obligation to be answerable for one’s own actions.^39^ Government policies and media narratives on obesity have previously adopted a neoliberalist approach to food policies which promotes a notion of free markets and individual responsibility for health instead of advocating government regulations on the food system.^40,41^ In the recent UK Government Food Strategy,^42^ there is recognition of a shared responsibility between industry, government and individuals but much of the focus remains on individual behaviour change, which is more difficult for individuals experiencing socioeconomic disadvantage. Public health advocates who are dedicated to addressing dietary inequalities argue that responsibility has to be proportionally attributed to consumers on the basis of their capacity to act.^43^

 Consumers habitually shop in the same way and are also likely to have fewer time, financial or critical analysis resources to make informed decisions in the moment.^44^ Furthermore, there is a need to raise awareness about the commercial determinants of diet among consumers.^45^ Increasing awareness in this way could boost public support for government policies and lead to changes in social norms for shopping behaviours. Civil society groups such as Sustain and Obesity Health Alliance are campaigning and calling for bold Government action^11,46^ and academics can support these activities by conducting social experiments that explore values such as autonomy,^47^ and by improving how scientific evidence in this field is disseminated to the public.^48^

###  Strengths and Limitations

 To our knowledge this is the first study to explore consumers’ perspectives of the new promotion and placement legislation and to increase understanding of its potential impact before implementation. The findings can guide future evaluation of the legislation’s effectiveness and strategies to enhance public support for it. While equal numbers of participants from the intervention and control stores in the WRAPPED study were invited to take part in this qualitative study, greater numbers agreed from the control stores, however, no differences were seen in responses between participants from the intervention and control stores. Women of childbearing age were targeted in this study because they remain primarily responsible for household food-related responsibilities such as food shopping and cooking in many families.^49^ It is, however, possible that men, women at other phases of the lifecourse have different perspectives on the potential impact of the incoming legislation and future research would benefit from engaging with a more diverse population sample. Nevertheless, the participants interviewed represent a valuable sample of women from both more and less disadvantaged backgrounds, northern and southern regions of England and with and without children. This study recruited women who shopped at the collaborating discount supermarket. Many women also reported shopping at other stores, however, those who shop exclusively from mid-range and premium supermarkets, or other store types, may have different perspectives to the participants of this study. Another limitation of this study is that the question guide focused on anticipated effects of the legislation on shopping in supermarkets whereas the incoming legislation affects all retail stores with more than 50 employees that sell HFSS products in prominent locations including franchises and symbol group convenience stores, and non-food retailers who sell HFSS products. The likely impact of the legislation in retail settings other than supermarkets should be assessed in future research. The timing of these interviews conducted was during the first wave of the COVID-19 pandemic; however, the results show this had little impact on participants’ perceptions of the likely promotions and placement restrictions. Participants were given the opportunity to reflect upon and discuss the impact of the COVID-19 lockdown on their shopping behaviours prior to moving to questions about retail marketing strategies and the incoming legislation. Participants shared both positive and negative views about the incoming legislation and their views were validated with representatives from the WRAPPED PPI panel.

## Conclusion

 This study explored consumers’ perceptions of the likely impact of the incoming food placement and promotion legislation on their food shopping behaviours. While consumers were generally positive that the incoming legislation could reduce impulse buying of HFSS, they raised concerns that the high price of healthier foods could undermine the legislation’s benefits. Coupling the legislation with interventions to promote and reduce the costs of healthier products would go further to ensure its success. Furthermore, with the current cost of living crisis and the fact that those with the lowest food budgets tend to plan their shopping in detail, evaluations of the intended and unintended consequences of the legislation on health inequalities should explore the impact across a range of sociodemographic groups. Finally, initiatives to raise awareness about the influence marketing strategies have on consumer food choices, that play into consumer concerns for cost and autonomy, would be helpful to further increase consumer acceptance of, and support for, the legislation.

## Acknowledgements

 We are grateful to the women who participated in this study and thank the dedicated team of research administrative staff especially Calum Shand, Julie Coleman, and Sue Curtis for their administrative support and contributions.

## Ethical issues

 This study was approved by the University of Southampton Faculty of Medicine ethics committee (Ethics ID: 20986.A6), and abides by the Declaration of Helsinki, Research Governance Framework for Health and Social Care and Data Protection regulations.

## Competing interests

 Authors declare that they have no competing interests.

## Funding

 This research and the authors of this paper are supported by the following funding sources: National Institute for Health Research (NIHR) Public Health Research Programme (grant funding, 17/44/46), NIHR Southampton Biomedical Research Centre and the UK Medical Research Council (UKMRC). The views expressed in this publication are those of the authors and not necessarily those of the NHS, the NIHR, the UK Department of Health and Social Care or UKMRC.

## Supplementary files

Supplementary file 1. Semi-structured Interview Guide – Consumers.
