# Supplementary material for: Women Consumers’ Views on Legislation to Restrict Prominent Placement and Multibuy Promotions of High Fat, Sugar, and Salt Products in England: A Qualitative Perspective
Source: Int J Health Policy Manag. 2023 Sep 10;12:7597. doi: 10.34172/ijhpm.2023.7597 (PMC10590244; doi:10.34172/ijhpm.2023.7597)
Supplement: Supplementary file 1 — Semi-structured Interview Guide – Consumers. [file ijhpm-12-7597-s001.pdf]

Article title: Women Consumers' Views on Legislation to Restrict Prominent Placement and Multibuy Promotions of High Fat, Sugar, and Salt Products in England: A Qualitative Perspective

Journal name: International Journal of Health Policy and Management (IJHPM)

Authors' information: Preeti Dhuria[1]*, Sarah Muir[1], Wendy Lawrence[1,2], Emma Roe[3], Sarah Crozier[1,4], Cyrus Cooper[1,2], Janis Baird[1,2,4], Christina Vogel[5,1,2,4]

[1]Medical Research Council Lifecourse Epidemiology Centre, University of Southampton, Southampton General Hospital, Southampton, UK

[2]National Institute for Health Research Southampton Biomedical Research Centre, University of Southampton and University Hospital Southampton NHS Foundation Trust, Southampton, UK

[3]School of Geography and Environmental Science, University of Southampton, Southampton, UK

[4]NIHR Applied Research Collaboration Wessex, Southampton Science Park, Innovation Centre, Southampton, UK

[5]Centre for Food Policy, City, University of London, London, UK

***Correspondence to**: Preeti Dhuria, Email: pd@mrc.soton.ac.uk

Citation: Dhuria P, Muir S, Lawrence W, et al. Women consumers' views on legislation to restrict prominent placement and multibuy promotions of high fat, sugar, and salt products in England: a qualitative perspective**.** Int J Health Policy Manag. 2023;12:7597. doi:10.34172/ijhpm.2023.7597

Supplementary file 1. Semi-structured Interview Guide – Consumers.

MRC Lifecourse Epidemiology Unit
Southampton General Hospital
Southampton S016 6YD
Telephone: 023 8120 4186

The WRAPPED study

ID number: | | | | **Date:** d d m m y y   **Time:**

Semi-structured interview guide - Participants

Introduction

Hello, I'm *[name]* from the University of Southampton. I am one of the WRAPPED study researchers. Thank you for agreeing to me interviewing you today. Before we get started, I wanted to check that you have read the information letter and are still happy to take part?

Great. Before we get started, I'd just like to run through a few things with you, including this consent form *[Appendix 9 v3]*.

The aim of this interview is to learn how the COVID-19 pandemic has affected your food shopping habits. We are also keen to hear how the layout of products in stores influences your shopping and what can the government and retailers do to support healthier food choices. With your permission, we will audio-record our conversation and I will take some notes. The entire interview usually takes between 30-40 minutes, depending on how much you have to say. Do you have any questions?

SHOPPING DURING PANDEMIC

- How would you describe your food shopping patterns before the rise of the Covid-19 pandemic?
- How has the pandemic changed your shopping habits?
- How have you found shopping in-store or online with the current lockdown restrictions?
- How did you respond when you heard about other people stockpiling food?
- How have you and your family's eating, and cooking habits changed since the pandemic started?

I'd like to move on to hearing your thoughts about some of the marketing strategies that supermarkets use to sell their food products. Supermarkets generally use location of a food product (in store and on shelves), and price promotions to encourage customers to buy certain products.

PRODUCT PLACEMENT

- How tempted are you to buy products in prominent location? [i.e., i) at the store entrance, ii) on end of aisle displays, iii) at checkouts]
    - What type of food products do you usually buy in these locations?
- How do you think the location of products on the shelf affects your food shopping decisions?
    - What shelf location (bottom, mid, high) do you buy from most often?

PRICE PROMOTIONS

- How do price promotions, such as multibuy promotions like two for one offer or 50% extra for the same price, affect your food shopping decisions?
    - What type of food products do you usually buy on price promotion?

The British government is planning to bring in legislation to restrict some marketing strategies in supermarkets being used on unhealthy foods, in particular banning unhealthy foods from being located at the store entrance, end-of-aisles, and checkouts and from being on price promotion.

SUPPORTING HEALTHIER FOOD CHOICES

- What do you think about these restrictions being introduced (on high fat, sugar, and salt products)?
- How do you think these restrictions on unhealthy foods will affect your shopping habits?
- What products would you like to see in prominent locations or on price promotions in supermarkets?
- What responsibility do you think supermarkets and the government have to support customers to buy healthier foods?
    - What do you think is the role of individual, supermarkets, and government in supporting customers make healthier food choices?

End with:

- What else would you like to share, or ask me about?
- We will send you a voucher by post next week as a thank you for contributing to this research. Thank you for your time.
